# Phosphate-Solubilizing Microbiota of Compost Elicited with Different Silicon Oxide Nanostructures to Increase Their Mineralization and Solubilization Properties

**DOI:** 10.3390/microorganisms14030519

**Published:** 2026-02-24

**Authors:** María del Pueblito Guevara-Santana, Ramón Gerardo Guevara-González, Jesús Angole-Tierrablanca, Enrique Rico-García, Irineo Torres-Pacheco, Viviana Palos-Barba, Sergio de los Santos-Villalobos, Adrián Esteban Ortega-Torres

**Affiliations:** 1 Biosystems Engineering Group, Center of Applied Research in Biosystems (CARB-CIAB), School of Engineering, Autonomous University of Querétaro, Campus Amazcala, Carr, Amazcala-Chichimequillas Km 1.0, El Marqués 76265, Mexico; mary.guevara.santana@gmail.com (M.d.P.G.-S.); ramonggg66@gmail.com (R.G.G.-G.); jesus.a.angole.t@gmail.com (J.A.-T.); ricog@uaq.mx (E.R.-G.); irineo.torres@uaq.mx (I.T.-P.); 2 Division of Research and Postgraduate Studies, Faculty of Engineering, University Center, Autonomous University of Querétaro, Querétaro 76010, Mexico; viviana.pb@uaq.edu.mx; 3 Microbial Resource Biotechnology Laboratory, Technological Institute of Sonora, 5 de Febrero 818 Sur, Col. Centro, Ciudad Obregón 85000, Mexico; dlsantosv@gmail.com

**Keywords:** macronutrient, microorganism, recycling, nanotechnology, environmental sustainability

## Abstract

The overreliance on non-renewable phosphate fertilizers necessitates sustainable alternatives for phosphorus recycling in agriculture. This study aimed to characterize and enhance the metabolic activity of phosphate-solubilizing microorganisms isolated from compost by eliciting them with two distinct mesoporous silica nanoparticles: standard SBA-15-S and short-pore SBA-15-C. Bacterial strains with broad-spectrum P solubilization and mineralization capacities were isolated from the mesophilic phases of tomato greenhouse and cow manure composts. These isolates received treatment with nanoparticle concentrations of 0.1, 10, and 100 ppm. The results demonstrated that nanoparticle elicitation significantly altered microbial growth, solubilization halos on tricalcium phosphate, and the specific activity of acid, neutral, and alkaline phosphatases in a strain- and nanoparticle-dependent manner. Notably, SBA-15-C at 100 ppm consistently enhanced multiple P-recycling properties across several strains, including *Proteus* and *Myroides* species. Principal component analysis revealed distinct behavioral clusters between composting phases and isolation methods. The findings indicate that tailored silicon oxide nanostructures can serve as eustressors to modulate and enhance the P-solubilizing and mineralizing functions of compost-derived microbiota, offering a promising nanobiostimulation strategy for developing enhanced biofertilizers.

## 1. Introduction

Current agricultural production relies heavily on chemical fertilizers, which are imported in different quantities by almost all countries [1]. Therefore, their use in soil and plant fertility is one of the most important factors globally. Global fertilizer projections focus on nitrogen (N), phosphorus (P), and potassium (K), with a projected average annual increase of 1.4%, 2%, and 3.3%, respectively, in 2014 [2]. Fertilizers have played a key role in crises due to their critical link between fossil fuel and food prices, with food ultimately becoming 44% more expensive; in the current geopolitical situation, the increase has fluctuated annually from 125% to 17% [3,4].

Since 2014, phosphorus has appeared on the European Commission’s list of crucial raw materials for the global economy, as industry obtains it from the exploitation of some limited rock deposits of apatite and phosphorite [5]. The global phosphorus landscape is also under pressure from non-food uses, primarily in the production of biofuels and lithium batteries [6,7,8]. In the 2019 FAO report [9], the global phosphorus balance for 2022 was 771,000 kt; the Americas are seriously unbalanced, with a deficit of −5409 kt. In this global context, when farmers apply phosphorus to the soil, plants assimilate only 5–30% of it; precipitation leaches the remainder; or soil cations immediately immobilize it, making it inaccessible to subsequent plant uptake [10]. This phosphorus could be an alternative for addressing the crisis and reducing environmental degradation, and technological interventions could address soil property degradation and loss while restoring biogeochemical cycles [11,12].

The management of organic waste (OW) as biological fertilizers has been reported as an alternative to chemical fertilizers, although barriers arise due to uncertainty regarding nutritional content and soil sustainability [13,14]. The presence of phosphorus (P) in organic waste has been reported at high percentages, up to 92%, depending on the waste source [15]. Some of its main organic forms are phytate, nucleic acids, phospholipids, phosphoproteins, phosphate sugars, and coenzymes [16,17]. In the natural phosphorus cycle, these organic and immobile forms are transformed into inorganic forms, such as available orthophosphates, by phosphate-solubilizing microorganisms (PSMs) through their mineralization and solubilization processes [18]. PSMs are a multifaceted group that includes actinobacteria, bacteria, fungi, arbuscular mycorrhizae, and cyanobacteria, and they can hydrolyze organic and immobilized phosphorus, transforming it into soluble forms [19]. Additionally, these microorganisms can promote N and K recycling, produce phytohormones, and enhance plant resistance to stress and phytopathogens, thus constituting an important alternative for N:P:K supplementation and for agroindustry [20,21].

The presence of PSMs has been identified in all stages of composting processes [22,23,24]. Composting is a useful environmental technology for recycling OW under controlled conditions until a stable material rich in microbial activity, humic substances, and nutrients for crops is obtained [25,26]. Composting promotes the development of microbial metabolic pathways involved in nutrient recycling and humification, creating an environment conducive to identifying microorganisms and metabolites of interest in the current context of food security. In the traditional composting process, mature compost has not met plants’ inorganic phosphorus (Pi) requirements due to low conversion rates. Advances in increasing Pi have focused on the study of plant-based microorganisms (PBMs), leveraging the potential of their individual mechanisms and/or microbial consortia for additive or synergistic effects [27,28]. However, most research has only evaluated some of the solubilizing properties [29,30]. Ortega-Torres et al. [31] studied the mineralization of *P. aeruginosa* ATC, finding that this strain had a metabolism capable of producing a cocktail of acid, neutral, and alkaline phosphatases and phytases, with which, as a supernatant, it increased Pi by 95% when applied to mature compost.

Recently, the study of microbial elicitation has aimed to stimulate physiological responses to stressors, leading to the production of secondary metabolites or enzymes of biotechnological interest [32]. In the field of microorganism elicitation, SiO_2_ nanoparticles (SiO_2_-NPs) are increasingly recognized as stressors with beneficial effects on agricultural microorganisms [33,34]. In one study, researchers extracted 30 nm SiO_2_-NPs for the plant *Equisetum telmateia* and identified the strains *P. stutzeri* and *Mesorhizobium* sp. Their results demonstrate that applying 100 ppm of silica nanoparticles to soil with both strains increased P content by 75–80 kg ha^−1^ compared with 40 kg ha^−1^ in the control and increased the dry weight of cress roots and shoots [35]. Tian et al., [34] investigated the foliar application of SiO_2_-NPs to the leaves of pak choi (*Brassica chinensis* L.) grown on contaminated mine soil with cadmium, zinc, and plumb; the treatments do not have adverse effects on photosynthesis or plant biomass but have an effect on protecting foliar plumb absorption, drastically altering rhizosphere metabolites, increasing sugars and organic acids, increasing abundancy of beneficial microorganisms, and improving soil quality. Ferrusquía-Jimenez et al. [36] demonstrated that applying SiO_2_-NPs (100 ppm) to *Bacillus cereus*-Amazcala improved P solubilization and increased antioxidant enzymatic activity, acting as a eustressor without harming the bacteria.

In this work, we aim to characterize and promote the metabolic activity of PSM bacterial strains isolated from compost by applying two different types of SiO_2_-NP to determine whether the surface structure or pore diameter increases PSM and metabolite production and thus create a strategy to increase Pi for plants.

## 2. Materials and Methods

### 2.1. Isolation of P-Solubilizing Microorganisms

The samples were collected from the mesophilic phases I (M1) and II (M2) of the active composting zones, which are composed of waste from tomato greenhouses and cow manure, combined at a stable C/N ratio. The composting facility was located at the Amazcala Campus of the Autonomous University of Querétaro, El Marqués, Querétaro, Mexico, with the following coordinates: 20.74° N, −100.32° W. Compost piles of 1 m × 2 m and 1.5 m high were established, in triplicate.

The piles were watered every 3 days with 15 L of water to maintain moisture at approximately 60% of field capacity. Temperatures were recorded 48 h after watering at five points, dividing the total depth and width equally. Manual aeration was performed weekly for 12 weeks [37].

The mesophilic phase I (M1) was identified by recording the temperature on the first day, when it began, at around 17 °C, and it rose to around 40 °C around the 15th day, ending M1; and mesophilic phase II (M2) began in the 7th and 9th week, when the temperature began to drop from 40 °C to around 20 °C, this stage lasting 2 weeks. The compost piles were stratified into three depth sections (0–30 cm, 40–70 cm, and 80 cm) and sampled horizontally (left, center, and right positions) using a corer to collect 3 g samples per point. The compost samples were homogenized, and 1 g of compost was serially diluted in 9 mL of sterile saline solution, up to a dilution factor of 10^6^. From each sample, 1 mL was taken and inoculated on selective phosphorus Pikovskaya agar (PVK) containing tricalcium phosphate (TCP) (Ca_3_(PO_4_)_2_) as the sole P source [38]. Additionally, samples were inoculated in 3 selective media developed from p-nitrophenol phosphate as the sole P source (NBRIP (SYGMA-ALDRICH Co., St Louis, MO, USA)), each medium with its pH: 6 to test the presence of acid phosphatases (A), 7 for neutral phosphatases (N), and 9 for alkaline phosphatases (B), according by Tabatai and Bremner [39].

The grown colonies were reseeded on LB agar medium using the depletion streaking technique and incubated at room temperature until completely isolated cultures were obtained.

Strains isolated from M1 and M2 through each selective medium were subjected to a final test to evaluate their metabolic capacities related to P recycling for their selection as compost-isolated phosphate-solubilizing microorganisms (CIPSMs), which consisted of inoculating them in the media from which they were not isolated, for the entire spectrum of phosphorus solubilization and mineralization; for example: one strain isolated PVK (PCa) and was inoculated into acid A (PhoA), neutral N (PhoN), and basic B (PhoB), and so on; once the strains with their extensive P metabolism were selected and labeled as CIPSMs, a Gram stain was performed to classify the bacterial strains [40].

### 2.2. Synthesis and Characterization of SBA-15-S and SBA-15-C SiO_2_ Nanoparticles

The mesoporous material SBA-15 was obtained through the sol–gel method following the procedure described by Flodström and Alfredsson, using tetraethylorthosilicate (TEOS) as a silica precursor and the surfactant Pluronic as a structuring agent.

In the standard synthesis, 4.8 g of the P123 copolymer (PEG-PPG-PEG, Mn ~5800, Sigma Aldrich, Steinheim, Germany) was dissolved in a mixture of 112.5 mL of deionized water and 75 mL of 4 M HCl, maintaining constant stirring at 35 °C. Next, the surfactant was dissolved; TEOS (98%; Sigma Aldrich, Steinheim, Germany) was gradually added to the final volume of 11 mL, maintaining the same temperature and stirring conditions for 24 h to start the sol–gel process. As a result of this procedure, the SBA-15-S (standard) was obtained, characterized by textural properties of its hysteresis/pores in well-defined cylindrical pores, with a surface area of 759 m^2^ g^−1^, and pore diameter of 4.3 nm.

In one of the experimental variants, we adjusted the precursor quantity to 8 mL of TEOS to evaluate its effect on the properties of the final material [41]. This procedure produced SBA-15-C (short size), characterized by textural properties of its hysteresis/pores in the same well defined cylindrical pores, with an increase in the surface area of 1235 m^2^g^−1^, and the pore diameter of 5.6 nm.

After gel formation, the samples underwent a maturation treatment in closed polypropylene containers at 80 °C for 24 h. They were then cooled to room temperature, filtered to recover the solid, and dried first at room temperature and then at 110 °C for 18 h with a heating ramp of 2 °C/min. Finally, the materials were calcined at 550 °C for 4 h with an increment of 1 °C/min, thus eliminating the organic phase and obtaining only the mesoporous silica structure.

**Figure 1:**
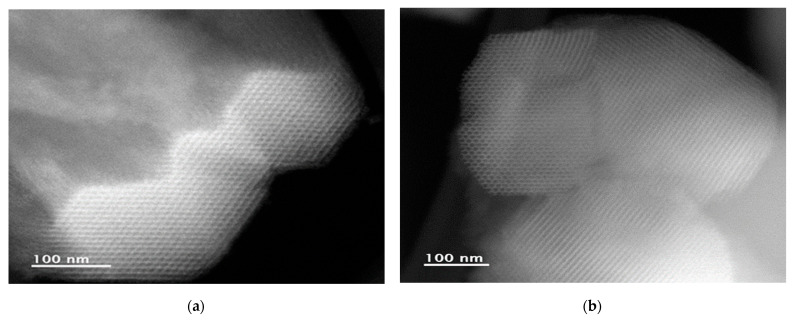
Scanning transmission electron microscopy for SBA-15 materials: (**a**,**b**) pore channels for SBA-15-S reference and modified SBA-15-C, respectively.

A Nanotech TEM JEOL JEM 2200FS+CS microscope provided morphological information about the materials through scanning transmission electron microscopy (STEM) (Figure 1).

### 2.3. Molecular Identification of P-Solubilizing Microorganisms

Genomic DNA was extracted from the 10 selected CIPSM isolates from liquid cultures in LB broth, following the Mahuku extraction protocol [28]. PCR was performed using the PCR master mix 2× from thermo scientific targeting 16s rRNA region with the following primers: forward 5′ [GCTTTTAGCTGTCGCTTGGA] 3′ and reverse 5′ [TGCATCTCTGCATACGTCAA] 3′ and the following program: 95 °C 10 min, 30 cycles of 95 °C 1 min, 50 °C 1 min, 72 °C 1 min, and the final extension of 72 °C 10 min.

Sanger sequencing was carried out at the National Laboratory of Agricultural, Medical, and Environmental Biotechnology of the IPICyT using the dideoxynucleotide method on the Genetic Analyzer 3130 sequencer (Applied Biosystems ABI Hitachi, Tokyo, Japan). Sequence analysis was performed on the National Center for Biotechnology Information (NCBI) website using nBLAST+ 2.17.0.

### 2.4. Elicitation of P-Solubilizing Microorganisms Isolated from Composting with Different SiO_2_ Nanostructures

The CIPSMs were incubated and activated in 15 mL Falcon tubes containing LB medium for 24 h at 30 °C with shaking (200 rpm). For the SBA-15-S and SBA-15-C nanoparticle treatments, sterile 15 mL Falcon tubes were prepared at concentrations of 0.1, 10, and 100 ppm, with 0 ppm as a control, and filled with sterile LB medium. The CIPSMs were inoculated at 3% of their volume and incubated for 24 h at 30 °C and 200 rpm in a shaking incubator. Finally, we measured the specific mineralization activity of the PhoA, PhoN, and PhoB at 24 h and took samples for the MPN and solubilization zone analysis.

First, their growth was evaluated using the most probable number (MPN) method, performed through spread plating: 5 µL of the 10^−5^ dilution was deposited on the agar surface and spread with a sterile Drigalski loop. The assay was performed in triplicate, and measurements were taken every 24 h for 7 days [42]. For solubilization properties, we used a solid PVK (CaPO_3_) medium in 35 × 10 mm Petri dishes. A total of 5 µL of the treatments and the control were placed on the agar and incubated at 30 °C for 7 days; on the last day the solubilization halo was measured with a vernier caliper [43].

To evaluate the mineralization properties, NBRIP liquid (p-nitrophenol phosphate) was prepared, sterilized, and dispensed into sterile 1.5 mL Eppendorf tubes. The CIPSMs were then inoculated with the treatments and incubated for 24 h at 30 °C; the cultures were centrifuged at 5000 rpm for 5 min; and the supernatant was used to measure the presence of the acidic (A), neutral (N), and basic (B) phosphatase (Pho) enzyme units. One milliliter of supernatant was measured at 420 nm using a Thermo Scientific SkyHigh Multiscan spectrophotometer (Waltham, MA, USA) to determine Pi. A standard p-nitrophenol curve was used to determine the enzyme unit [44]. One unit (U) of enzyme was defined as the amount required to produce 1 µmol of inorganic orthophosphate per milliliter per minute under assay conditions. A sample of the supernatant was taken to measure its protein content using the Bradford method [45].

The enzyme activity unit (U/mg of protein) was expressed as (U), the amount of enzyme required to produce 1 μmol of inorganic orthophosphate per milliliter per minute under the assay conditions, per mg of protein.

### 2.5. Statistical Analysis

All tests were performed in triplicate to ensure the reliability of the results.

The data obtained were statistically analyzed using analysis of variance (ANOVA) to determine the significance of the differences between treatments, and the results were further analyzed using Tukey’s test (*p* = 0.90), performed with R version 4.5.1 [46].

In the results, we present only the CIPSMs figures that showed significant differences compared to the control.

Additionally, the principal component analysis (PCA) was performed using RStudio with R version 4.5.1.

## 3. Results

### 3.1. Isolation and Identification of CIPSMs

A total of 20 PSM strains were isolated from the mesophilic phases M1 and M2 of composting, nine from M1 and eleven from M2. Specifically, 55% of the isolated strains belonged to the solubilizing metabolism (PCa) and 45% to the mineralizing metabolism (Pho). In M1, six solubilizing strains and one strain each were obtained from the PhoA and PhoB. In M2, the number of solubilizing strains decreased to five, while the number of mineralizing strains increased to eight, of which four were isolated from the PhoB, two from the PhoA, and two from PhoN.

Testing the total solubilization and mineralization properties of these isolates reduced the CIPSMs to the levels shown in Table 1.

### 3.2. Study of Elicited CIPSMs with Nanoparticles SBA-15-S and SBA-15-C

**Figure 2:**
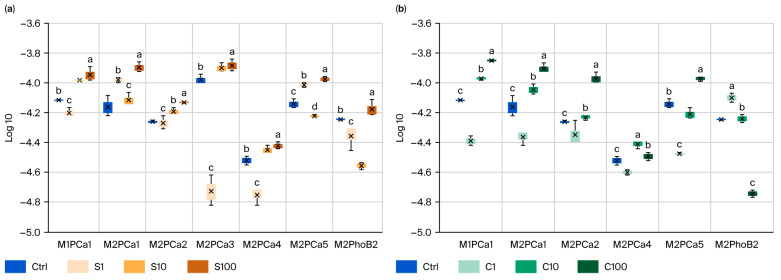
The effect of SiO_2_-NPs on CIPSM growth change: (**a**) CIPSMs elicited with SBA-15-S (S), (**b**) CIPSMs elicited with SBA-15-C (C). Different letters indicate significant differences according to Tukey’s test (α = 0.1). The figure presents only the CIPSMs that showed significant differences compared to the control.

The results of this elicitation study on CIPSMs revealed the importance of complementing them with several specific tests related to P recycling, as some tests showed a positive effect. In contrast, in others, it was negative, both among the strains and the size of the SBA-15, regarding in vitro growth, mineralization, and/or solubilization properties, as described in this section. Figure 2 shows the growth results. We observed seven strains with significant effects on SBA-15-S(S) compared to the control group (Ctrl), and six with significant effects on SBA-15-C(C), indicating a greater response to the CFU concentration with C100 in strain M1PCa1 (1.4 × 10^5^). In this test with SBA-15-S, six PCa-solubilizing strains had a positive effect. Strain M2PCa3 with S-100 exhibited the highest concentration of 1.3 × 10^5^ CFU, twice that of the control. M1PCa1, M2PCa2, and M2PCa5 with S-100 showed a considerable increase compared to the control group, with levels four times higher (Figure 2a). Regarding SBA-15-C, five PCa-solubilizing strains were observed. Most PCa strains showed an effect on elicitation with C100; these were M1PCa1, M2PCa1, MPCa2, and M2PCa5, with increases three or more times compared to that of the control. Strain M2PhoB2 was the only mineralizing strain to show a significant increase compared to the control with S100 (2 times higher) and with C1 (3.5 times higher).

**Figure 3:**
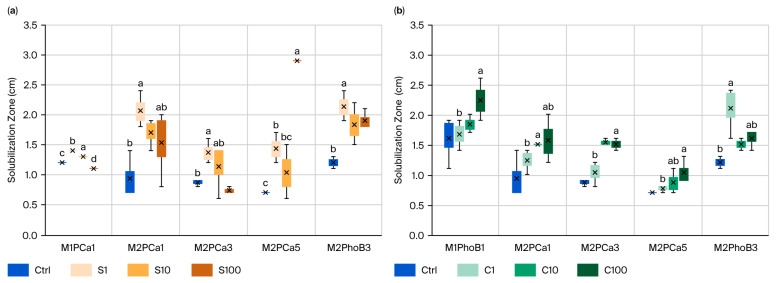
The effect of SiO_2_-NPs on CIPSM solubilization (CaPO_3_): (**a**) CIPSMs elicited with SBA-15-S (S), (**b**) CIPSMs elicited with SBA-15-C (C). Different letters indicate significant differences according to Tukey’s test (α = 0.1). The figure presents only the CIPSMs that showed significant differences compared to the control.

In the test on the microbiological solubilization properties, elicitation with S and C positively affected five CIPSM strains. Strain M1PCa1 was replaced in C with M1PhoB1; the other three strains, M2PCa1, M2PCa3, and M2PCa5, were solubilizing and remained unchanged from the CFU test. Two basic mineralizing strains, M1PhoB1 and M2PhoB3, were also added and maintained the effect with both SBA-15 (Figure 3). In the SBA-15-S elicitation effect, we obtained the best results with strain M2PCa5, which had the most significant impact on solubilization with S10 (314%), followed by strains M2PCa1 and M2PhoB3, both with S1, which showed a substantial effect of 114% and 70% more, respectively (Figure 3a). The elicitation effect with SBA-15-C showed less effect in the solubilization test, where the mineralizing strains showed the best effect. M1PhoB1 and M2PhoB3 increased by 40% and 75% with C100, respectively (Figure 3a). The C100 concentration had almost the same effect on the strains as in the CFU test. It is worth noting that, fortunately, most strains responded to the elicited effect; however, some responded only at a specific nanoparticle concentration, and the same strains responded regardless of the isolation method.

**Figure 4:**
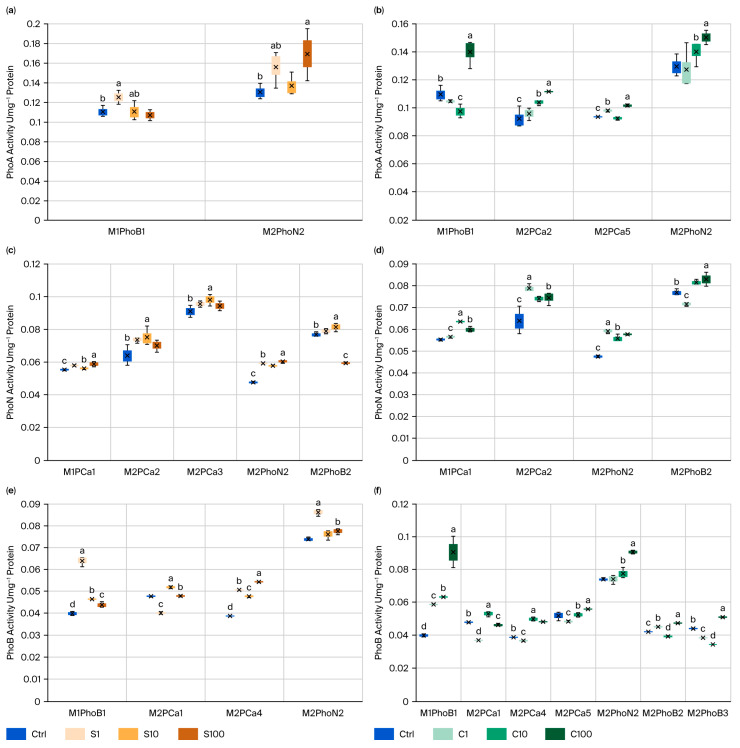
The effect of SiO_2_-NPs on CIPSM mineralization through phosphatases: (**a**) CIPSMs with PhoA elicited with SBA-15-S (S), (**b**) CIPSMs with the PhoA elicited with SBA-15-C, (**c**) CIPSMs with PhoN elicited with S, (**d**) CIPSMs with the PhoN elicited with C, (**e**) CIPSMs with the PhoB elicited with S, (**f**) CIPSMs with the PhoB elicited with C. Different letters indicate significant differences among treatments per strain according to Tukey’s test (α = 0.1). The figure presents only the CIPSMs that showed significant differences compared to the control.

In the elicitation of mineralizing properties through the specific phosphate-solubilizing activity A, N, and B (Figure 4) and in the elicitation for the PhoA with SBA-15-S, only two strains showed significance: first, M2PhoN2 with a 30% increase in S100, and second, M1PhoB1 with a 13% increase in S1. These strains showed significant responses in acid phosphatase (PhoA), exhibiting enzymatic activity distinct from that of the strains from which N and B were isolated, respectively (Figure 4a). These strains remained active under the SBA-15-C elicitation, with the most representative strains being those stimulated with C100. M2PhoN2 showed the highest activity, with a 16% increase, followed by M1PhoB1 with a 24% increase. This supports the conclusion that these strains were representative and exhibited solubilizing properties with this NP and concentration, as observed (Figure 4b).

The results of the specific phosphate-solubilizing activity (PhoN) elicitation with SBA-15-S showed significant differences (Figure 4c). Five CIPSMs showed substantial differences: three were solubilization and two were mineralization, responding better to S10. M2PhoN2 with S10 showed the most significant increase in elicitation compared to the control (27%) but presented one of the lowest solubilizations among the specific activities, along with M1PCa1. M2PCa3 with S10 showed the highest specific activity, 8% higher, followed by M2PhoB2, which appeared for the first time in this study and showed a 6% increase. In Figure 4d, the specific activity elicited with SBA-15-C did not show significant differences for M2PCa3, except in four CIPSMs. The results show, for this enzyme, a change in the observed effects according to the NP concentration: M2PhoB2 with C100 showed the highest specific phosphate-solubilizing activity with an 8% increase, followed by M2PCa3 with C1 increasing by 23%, then M1PCa1 with C10 rising by 15%, and finally M2PhoN2 with C1 rising by 25%. We note that the positive effects of the NPs on CIPSMs are evident. In this specific assay, the strains with the best PhoN properties were those that showed the highest elicited activity.

The latest results for specific phosphate-solubilizing activity (PhoB) (Figure 4e) show that SBA-15-S elicited an effect in four CIPSM strains. Strain M2PhoN2 stimulated with S-1 showed the highest concentration of PhoB activity, increasing by 17%. This was followed by M1PhoB1, with S-1 rising by 61%. Finally, we present two new strains, M2PCa1 and M2PCa4, with significant solubilizing activity upon S10 stimulation. Similarly, in the elicitation with SBA-15-C, the significant specific phosphate-solubilizing activity was maintained in the same mineralizing strains, M1PhoB1 and M2PhoN2, throughout this experiment (Figure 4f). In this assay, we detected the highest number of CIPSM strains, totaling seven. The assay included all four mineralizing CIPSM strains (M1PhoB1, M2PhoN2, M2PhoB2, and M2PhoB3), which were all stimulated with C100. M1PhoB1 showed the best specific activity, with a 127% increase, followed by M2PhoN2 with C100, which showed a 22% increase. This strain exhibited good overall property B across all treatments. Among the solubilizing strains that affect the PhoB, M2PCa1 with C10 and M2PCa5 with C100 showed effects of 11% and 8%, respectively. The last strain, M2PCa4, showed no enzyme activity but an impact of 28% with C10. The mineralizing strains maintained good results for the PhoA and PHoB.

### 3.3. Principal Component Analysis

**Figure 5:**
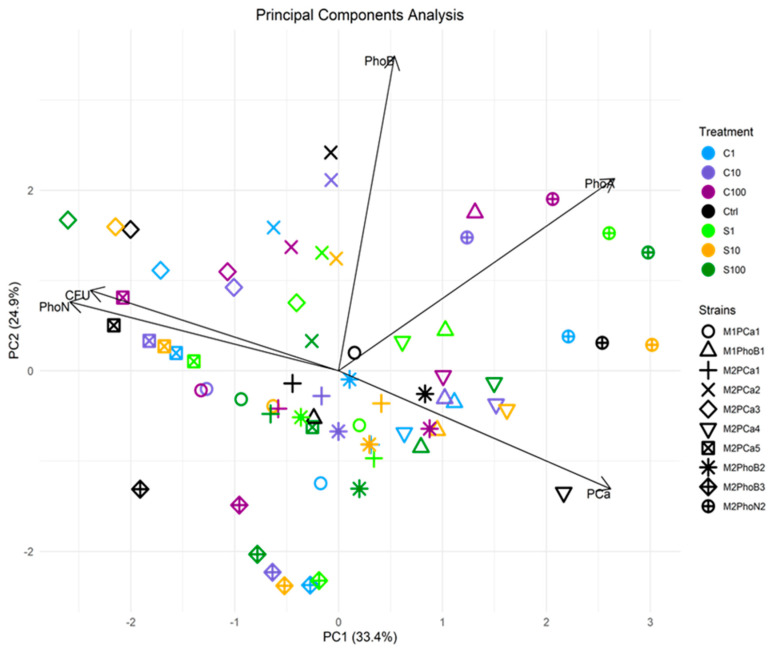
Principal components analysis. M1 and M2 represent the mesophilic phases from which the strains were obtained, while B, N, and PCa indicate the isolation methods applied to each strain. Treatments C1, C10, and C100 correspond to the administration of short nanoparticles, whereas S1, S10, and S100 represent treatments with simple nanoparticles.

Principal component analysis (PCA) was conducted to compare the results of strains derived from mesophilic phases 1 and 2, along with their respective isolation methods, against measurements of the solubilization halo (PCa) and enzymatic activities—neutral (PhoN), acidic (PhoA), and basic (PhoB)—as well as colony-forming units (CFU). Principal components 1 and 2 together accounted for 58.3% of the total variance (Figure 5).

This analysis allows us to describe the effects of elicitation with SiO_2_ nanoparticles SBA-15-S (S) and SBA-15-C (C) on the eustresic or dysstresic effects in CIPSMs, revealing behaviors and patterns in the alteration of their solubilizing properties for microorganisms. In summary, the previous tests showed a significant increase in the mineralizing and solubilizing properties of the selected CIPSM strains with the C100 treatment. At the same time, the behavior with S was more erratic. Individually, the strains that showed significant and consistent properties were, firstly, M2PhoN2 in the PhoB with S1, in the PhoA with S10, and in C100, exhibiting mineralization pathways completely different from where it was isolated and dominating in the PhoA mineralizing property; followed by M1PhoB1 in the PhoB and in the PCa with C100, remaining consistent with the SBA-15-C elicitation, both in the test of its isolation and in the solubilizing property. The strains with the highest results were M2PhoB2 in the PhoN with C100, M2PCa3, which had similar properties to the PhoN with S10, and, for the solubilization results, M2PCa5 in the PCa with S100; these were the significant phosphorus solubilization results for recycling.

The analysis revealed a strong positive correlation between UFC and PhoN, while it showed an inverse correlation with the PCa. No correlation was found with the PhoA and PhoB, although these showed a slight positive correlation. When comparing phases M1 and M2, as well as the CIPSM isolation method, the overall behavior of M1 reveals a marked difference in behavior according to isolation strain. The M1PCa1 isolation control appears related to the PhoA, and the effect of nanostructures on its solubilizer properties shows partial to left-down behavior with S and C on 10 and 100, oriented to UFC-PhoN, and central-bottom behavior from S1 to C1. For strain M1PhoB1, the control was located in the lower central area, while stimulation shifted the profile rightward toward the PCa and the PhoA. This shift formed a near-linear trajectory where S1, C1, and C10 clustered near the PCa, and S10 and C100 extended toward the PhoA, indicating the likely origin of the significant C100 results. In M2, strains M2PCa1 and M2PhoB2 formed a central cluster. For strain M2PCa2, both the control and treatments shifted towards the PhoB. Strain M2PCa3 shifted towards the UFC-PhoN axis, while the control for M2PCa4 positioned near the top of the PCa. In M2PCa5, the control located near the UFC-PhoN axis; however, its C100 treatment showed a strong correlation with UFC.

The isolated mineralization strain M2PhoB3 exhibited high variability in its behavior, similar to that of M2PhoN2, where a correlation was observed between C100 and PhoA, making it one of the best-performing strains. The effects of the isolation method indicate erratic behavior, sometimes directed towards the vectors, which is of microbial ecology. This is a result of the composting process and is related to the bioprospecting of these relationships to increase the efficiency of P release.

## 4. Discussion

Composting of agro-industrial organic waste is a valuable source for isolating phosphate-solubilizing microorganisms (PSMs) [47]. In Zhu et al. [48], 57 solubilizing PCa strains were isolated from compost, and the stress-tolerant yeast isolate FL7, identified as Pichia farinose, was selected for further studies. In another report, Kour et al. [49] isolated 15 bacteria from municipal solid waste compost and selected 4 that demonstrated solubilizing properties, with efficiency rates ranging from 70% to 85%. The diachronic study identifies microorganisms isolated from compost, highlighting the importance of defining the phases of the composting process to bioprospect composting and addressing national and international environmental and agricultural challenges. Similarly, the definition of phosphate-solubilizing strains, which have been named based on specific properties, is also relevant, and it is interesting to expand this classification for potential uses. This allows for future microbiological constructs to improve phosphorus recycling. 

Swain et al. [50] isolated five strains of *Bacillus subtilis* from composted cow manure. They discovered that these strains not only solubilized NBRIP but also produced the PhoA and PhoB. In this investigation, we isolated 20 strains from the mesophilic phase of cow manure compost to identify those with broad-spectrum phosphorus (P) recycling properties. Only 10 of these strains can solubilize the PCa and mineralize it across the entire pH range, and they contain the PhoA, PhoN, and PhoB; therefore, they are designated CIPSMs. In Vaz-Moreira et al. [51], the heterotrophic microbial diversity was isolated from five composts: chicken manure (CP), sewage sludge (SS), municipal solid waste (MSW), and two domestic composts (DC, domestic waste; VC, vermicompost). Gram-positive bacteria of the phylum Firmicutes predominated in all composts, with the genus Bacillus present in all of them, followed by Gram-negative bacteria of the phylum Proteobacteria. Our results identifying CIPSM in M1 are consistent with the descriptions of Proteobacteria as the most representative bacteria in M1 due to their beneficial functions in moisture retention and nutrient recycling [26,52]. However, in our results, M2 was the most representative, with six strains, and within the phylum Bacteroidetes, with two *Myroides* strains. Li et al. [53] reported that Proteobacteria, Firmicutes, Actinobacteria, Bacteroidetes, and *Chloroflexi* are the dominant bacterial phyla during composting. This finding demonstrates the resilience and adaptability of these phyla.

These identified CIPSM strains are directly related to those that cause urinary tract infections (UTIs). Genomic support is essential to complement and accurately assess their safety, as is the case with antibiotic-resistant strains (ARSs) [54]. Thanks to the scientific community, the reference genomic database is evolving, improving the accuracy of these detection tools and allowing access to new native microbial strains, such as those used in this research, which may be essential for maintaining recycling phosphorus processes and contributing to climate change regulation [55]. Furthermore, support from molecular techniques, which provide a more accurate picture of the microecological processes occurring in situ during composting, such as genotypic characterization by RAPD and REP, was used to group bacterial isolates by genetic patterns, compare diversity between composts, and apply metagenomics to analyze functional genes related to the P cycle [56,57]; these approaches will allow us to develop strategies for addressing the challenges of agricultural sustainability. It is necessary to begin expanding biosafety protocols for microorganisms used in agriculture to determine how to use them safely, either directly or as metabolites, and, as in this research, to enhance their phosphorus solubilization potential through elicitation with NPs.

We wanted to highlight multiple tests to demonstrate microbial preferences (according to the metabolic property analyzed) beyond the isolation method and show that these preferences can synergize with specific sizes or types of silicon oxide nanoparticles. The established tests proved to be adequate for the proposed elicitation; in other words, this occurs in microbial communities and follows the same rules of hormesis, such as the specific dose rule, the eustress rule, the activation and potentiation of the biological system, the independent response rule, and the specificity of physicochemical factors [36,58,59]. The CIPSMs in the experiments exhibited distress and eustress behaviors depending on the SBA-15 and the test. It is interesting to highlight how nanobiomaterial engineering influences biological molecules. Nabika and Unoura [60] indicate how, in a model cell membrane, the interaction with polyoxometalate (POM) nanoparticles causes lipids to rearrange themselves to adapt to the curvature of the nanoparticle, where nanoscale adsorption at the cell–membrane interface will vary depending on the size, charge, and surface state. This confirms that the use of silicon oxide NPs through the sol–gel method has achieved eustressor results by interacting with the building blocks of the cell to be modified, protecting its internal components, and promoting the desired overstimulation in the PGPM and CIPSMs [51], as well as that the size of SBA-15-S and SBA-15-C interacted in a contrasting way in the responses sought.

This result leads us to characterize the M1 strains by their rapid, active degradation of simple molecules and plant biomass [59,61], their broad phosphorus-recycling properties, and their positive response to NP elicitation, making M1PhoB1 a potential candidate for applications. CIPSM isolates from M2 exhibit variation, with six strains associated with the best activities: three isolated from the PCa-solubilizing medium and three from the mineralizing medium (two for B and one for N). Notably, the M2PhoN2 strain dominated the PhoA enzyme with S100 and C100, followed by the PhoB enzyme with S1 in the first place and C100 in the second. This strain exhibits potential in elicited properties A and B, distinct from the site where we isolated it in N, similar to what occurred with M1PhoB1 in M1. In M2, the Pca-isolated strains included M2PCa3, which shows potential in three evaluated solubilizing properties; with C100, it ranks 2nd in the solubilization zone, and with C100 in the PhoA enzyme, it ranks 4th. This strain in the PhoN enzyme showed results with S10, ranking 1st. Here, it is evident that the isolation medium does not determine the potential properties of the microorganisms. We selected this strain for its enhanced and significant mineralizing properties of N and A. The results indicate that mineralizing strains, independent of the isolation phase, are candidates for further research due to their consistency and significance in this study. Similarly, solubilizing strains of M2 exhibit broad mineralization and solubilization properties of P.

The properties developed by PSMs are directly influenced by external factors, starting with a critical initial signal: the concentration of Pi (orthophosphate) in the environment. PSMs adapt to phosphate levels by inducing mineralization under deficiency or repressing enzymes under excess. Production peaks illustrate this: *E. coli* and *P. vulgaris* produced maximum phosphatase at 0.1 M Pi, while *Salmonella* showed inducible activity at levels up to 0.5 M Pi [62]. The external presence of specific organic substrates acts as a signal that modulates Pho activity in *Serratia* sp., which uses its PhoA to aid in the utilization of glycerol-2-phosphate, or as in *Yersinia enterocolitica* and *Enterobacter cloacae*, where glycerophosphate (G3P) inhibited enzyme production [63]. The elicitation process with NPs reproduced the properties developed by the PSMs, which were induced by a specific external organic substrate (S and C) and by the concentration of Pï in the external environment, thus verifying the elicitation of its mineralizing property.

The solubilizing properties of PSMs link directly to pH changes, a key environmental determinant. These signaling changes release protons (H^+^) and low molecular weight organic acids, which dissolve phosphates and chelate metal cations, releasing Pi ions [48,64]. These properties are also directly related to other properties of the PGPRs, supporting the existence of synergy between NPs and CIPSMs as a eustress effect and suggesting that this could support the development of nanobiofertilizers and/or the use of metabolites elicited in vitro. Any such development should be subject to biosafety investigations. However, we believe that it is feasible to conduct a study on the elicitation of microorganisms and their application to current challenges, such as bioremediation, to act as a defense against heavy metal toxicity. These microorganisms act as cofactors or chemical inhibitors that can modulate the periplasmic acid phosphatase activity characteristic of these PSMs, a key enzyme in the bioprecipitation process of metals as insoluble phosphates.

## 5. Conclusions

This study demonstrates that elicitation with mesoporous silica nanoparticles (SBA-15) is an effective strategy for modulating and enhancing the activity of phosphate-solubilizing microorganisms (PSMs) isolated from compost. The structure and pore diameter of the nanoparticles (SBA-15-S vs. SBA-15-C) differentially influenced microbial growth, calcium phosphate solubilization, and specific enzymatic activity (acid, neutral, and alkaline phosphatases) depending on the bacterial strain and the applied concentration. Treatments with SBA-15-C at 100 ppm (C100) showed the most consistent and positive effects, significantly improving mineralization and solubilization properties in several strains, especially those with mineralizing metabolism, such as *Proteus* sp. and *Myroides* sp. This research proposes the design of improved microbial consortia, the application of concentrated metabolites via microbial elicitation, and the use of native microorganisms.

## Figures and Tables

**Table 1:** CIPSM strains related to all metabolic properties involved in phosphorus and their molecular identification.

Strain	Gram	Identification	% Identity	GenBank Accession No.
M1PCa1	+	*Proteus terrae*	98.93	SAMN54431734
M1PhoB1	-	*Proteus mirabilis*	91.93	SAMN54234480
M2PCa1	+	*Proteus mirabilis*	99.52	SAMN54431735
M2PCa2	-	*Myroides odoratimimus*	80.25	SAMN54431740
M2PCa3	-	*Serratia* sp.	26.58	SAMN54431737
M2PCa4	-	*Serratia* sp.	91.14	SAMN54431738
M2PCa5	-	*Proteus mirabilis*	99.1	SAMN54431739
M2PhoB2	+	*Myroides* sp.	80.59	SAMN54431736
M2PHoB3	-	*Alcaligenes faecalis*	84	SAMN54431741
M2PhoN2	-	*Proteus* sp.	85.2	SAMN54431742

## Data Availability

The original contributions presented in this study are included in the article. Further inquiries can be directed to the corresponding author.

## Abbreviations

The following abbreviations are used in this manuscript:

P	phosphorus
OW	organic waste
PSM	phosphate solubilization microorganisms
Pi	inorganic phosphorus
SiO_2_	silicon oxide
NPs	nanoparticles
M1	mesophilic phase I
M2	mesophilic phase II
C	carbon
N	nitrogen
PVK	Pikovskaya agar
TCP	tricalcium phosphate
A	acid phosphatases
N	neutral phosphatases
B	alkaline phosphatases
CIPSM	compost-isolated phosphate-solubilizing microorganisms
PCa	microorganism isolated with PVK or with TCP solubilization property
PhoA	microorganism isolated with A or with A mineralization property
PhoN	microorganism isolated with N or with N mineralization property
PhoB	microorganism isolated with B or with B mineralization property
S	nanoparticle mesoporous standard SBA-15-S
C	nanoparticle mesoporous short size SBA-15-C
CFU	colony-forming unit
MPN	most probable number
Pho	phosphatase enzyme
PCA	principal component analysis

## References

[1] Cruz-Cárdenas C.I., Zelaya-Molina L.X., Sandoval-Cancino G., Santos-Villalobos S.d.l., Rojas-Anaya E., Chávez-Díaz I.F., Ruíz-Ramírez S. (2021). Using microorganisms for a sustainable agriculture in Mexico: Considerations and challenges. Rev. Mex. Cienc. Agrícolas.

[2] Food and Agriculture Organization of the United Nations (2012). Current World Fertilizer Trends and Outlook to 2016.

[3] Gnutzmann H., Piotr S. (2016). Fertilizer fuels food prices: Identification through the oil-gas spread. SSRN.

[4] Hebebrand C., Debucquet D.L. (2023). High Fertilizer Prices Contribute to Rising Global Food Security Concerns. https://www.ifpri.org/blog/high-fertilizer-prices-contribute-rising-global-food-security-concerns/.

[5] Saeid A., Prochownik E., Dobrowolska-Iwanek J. (2018). Phosphorus Solubilization by Bacillus Species. Molecules.

[6] Rittmann B.E., Mayer B., Westerhoff P., Edwards M. (2011). Capturing the lost phosphorus. Chemosphere.

[7] Spears B.M., Brownlie W.J., Cordell D., Hermann L., Mogollón J.M. (2022). Concerns about global phosphorus demand for lithium-iron-phosphate batteries in the light electric vehicle sector. Commun. Mater..

[8] Banerjee S., Pufahl P.K., Dare S. (2024). Igneous Rock Phosphate: Ore Grades, Concentrates, and Mining Operations Around the World.

[9] FAO (2019). World Fertilizer Trends and Outlook to 2022.

[10] Mandal A., Sarkar B., Mandal S., Vithanage M., Patra A.K., Manna M.C., Kuhl M., Butterworth-Hinemann L. (2020). Impact of agrochemicals on soil health. Agrochemicalss Detection, Treatment and Remediation.

[11] Smith O.M., Cohen A.L., Reganold J.P., Jones M.S., Orpet R.J., Taylor J.M., Crowder D.W. (2020). Landscape context affects the sustainability of organic farming systems. Proc. Natl. Acad. Sci. USA.

[12] Zaman Q.U., Zaman S., Zhao Y., Rasool S.F., Qamar R. (2025). Policy Strategies for Sustainable Urban Development in the 21st Century: Fresh Empirical Evidence in Global Framework. Sustain. Dev..

[13] Thomas C.L., Acquah G.E., Whitmore A.P., McGrath S.P., Haefele S.M. (2019). The Effect of Different Organic Fertilizers on Yield and Soil and Crop Nutrient Concentrations. Agronomy.

[14] Chew K.W., Chia S.R., Yen H.-W., Nomanbhay S., Ho Y.-C., Show P.L. (2019). Transformation of Biomass Waste into Sustainable Organic Fertilizers. Sustainability.

[15] Sharpley A., Moyer B. (2000). Phosphorus forms in manure and compost and their release during simulated rainfall. J. Environ. Qual..

[16] Tamburini F., Pfahler V., Bünemann E.K., Guelland K., Bernasconi S.M., Frossard E. (2012). Oxygen isotopes unravel the role of microorganisms in phosphate cycling in soils. Environ. Sci. Technol..

[17] Tian J., Ge F., Zhang D., Deng S., Liu X. (2021). Roles of phosphate solubilizing microorganisms from managing soil phosphorus deficiency to mediating biogeochemical P cycle. Biology.

[18] Nash K.L., Allen C.R., Angeler D.G., Barichievy C., Eason T., Garmestani A.S., Sundstrom S.M. (2014). Discontinuities, cross-scale patterns, and the organization of ecosystems. Ecology.

[19] Kalayu G. (2019). Phosphate solubilizing microorganisms: Promising approach as biofertilizers. Int. J. Agron..

[20] Rawat P., Das S., Shankhdhar D., Shankhdhar S.C. (2021). Phosphate-solubilizing microorganisms: Mechanism and their role in phosphate solubilization and uptake. J. Soil Sci. Plant Nutr..

[21] Silva L.I.d., Pereira M.C., Carvalho A.M.X.d., Buttrós V.H., Pasqual M., Dória J. (2023). Phosphorus-Solubilizing Microorganisms: A Key to Sustainable Agriculture. Agriculture.

[22] Antoun H. (2012). Beneficial microorganisms for the sustainable use of phosphates in agriculture. Procedia Eng..

[23] Wei Y., Zhao Y., Wang H., Lu Q., Cao Z., Cui H., Wei Z. (2016). An optimized regulating method for composting phosphorus fractions transformation based on biochar addition and phosphate-solubilizing bacteria inoculation. Bioresour. Technol..

[24] Oliveira V.C., Damasceno F.A., Oliveira C.E.A., Ferraz P.F.P., Ferraz G.A.S., Saraz J.A.O. (2019). Compost-Bedded Pack Barns in the State of Minas Gerais: Architectural and Technological Characterization. Agron. Res..

[25] Sayara T., Basheer-Salimia R., Hawamde F., Sánchez A. (2020). Recycling of organic wastes through composting: Process performance and compost application in agriculture. Agronomy.

[26] Aguilar-Paredes A., Valdés G., Araneda N., Valdebenito E., Hansen F., Nuti M. (2023). Microbial community in the composting process and its positive impact on the soil biota in sustainable agriculture. Agronomy.

[27] Emami S., Alikhani H.A., Pourbabaee A.A., Etesami H., Motasharezadeh B., Sarmadian F. (2020). Consortium of endophyte and rhizosphere phosphate solubilizing bacteria improves phosphorous use efficiency in wheat cultivars in phosphorus deficient soils. Rhizosphere.

[28] Santoyo G., Guzmán-Guzmán P., Parra-Cota F.I., Santos-Villalobos S.D.L., Orozco-Mosqueda M.D.C., Glick B.R. (2021). Plant growth stimulation by microbial consortia. Agronomy.

[29] Sharma S.B., Sayyed R.Z., Trivedi M.H., Gobi T.A. (2013). Phosphate solubilizing microbes: Sustainable approach for managing phosphorus deficiency in agricultural soils. SpringerPlus.

[30] Amri M., Mateus D., Gatrouni M., Rjeibi M.R., Asses N., Abbes C. (2022). Co-inoculation with phosphate-solubilizing microorganisms of rock phosphate and phosphogypsum and their effect on growth promotion and nutrient uptake by ryegrass. Appl. Biosci..

[31] Ortega-Torres A.E., Rico-García E., Guzmán-Cruz R., Torres-Pacheco I., Tovar-Pérez E.G., Guevara-González R.G. (2021). Addition of phosphatases and phytases to mature compost to increase available phosphorus: A short study. Agronomy.

[32] Guan N., Li J., Shin H.D., Du G., Chen J., Liu L. (2017). Microbial response to environmental stresses: From fundamental mechanisms to practical applications. Appl. Microbiol. Biotechnol..

[33] Rangaraj S., Gopalu K., Muthusamy P., Rathinam Y., Venkatachalam R., Narayanasamy K. (2014). Augmented biocontrol action of silica nanoparticles and Pseudomonas fluorescens bioformulant in maize (*Zea mays* L.). RSC Adv..

[34] Tian L., Shen J., Sun G., Wang B., Ji R., Zhao L. (2020). Foliar application of SiO_2_ nanoparticles alters soil metabolite profiles and microbial community composition in the pakchoi (*Brassica chinensis* L.) rhizosphere grown in contaminated mine soil. Environ. Sci. Technol..

[35] Boroumand N., Behbahani M., Dini G. (2020). Combined effects of phosphate solubilizing bacteria and nanosilica on the growth of land cress plant. J. Soil Sci. Plant Nutr..

[36] Ferrusquía-Jiménez N.I., González-Arias B., Rosales A., Esquivel K., Escamilla-Silva E.M., Ortega-Torres A.E., Guevara-González R.G. (2022). Elicitation of *Bacillus cereus*-Amazcala (*B.c*-A) with SiO_2_ nanoparticles improves its role as a plant growth-promoting bacteria (PGPB) in chili pepper plants. Plants.

[37] Alavi N., Daneshpajou M., Shirmardi M., Goudarzi G., Neisi A., Babaei A.A. (2017). Investigating the efficiency of co-composting and vermicomposting of vinasse with the mixture of cow manure wastes, bagasse, and natural zeolite. Waste Manag..

[38] Wang C., Cui J., Yang L., Zhao C.A., Wang T., Yan L., Liu S. (2018). Phosphorus-release dynamics by phosphate solubilizing actinomycetes and its enhancement of growth and yields in maize. Int. J. Agric. Biol.

[39] Tabatabai M.A., Bremner J.M. (2005). Use of p-nitrophenyl phosphate for assay of soil phosphatase activity. Soil Biol. Biochem..

[40] Smith A.C., Hussey M.A. (2005). Gram Stain Protocols.

[41] Palos-Barba V., Mendoza R.N., Millan-Malo B.M., Aguilar-Franco M., Peza-Ledesma C., Rivera-Muñoz E.M. (2024). SBA-15 with short-sized channels modified with Fe_2_O_3_ nanoparticles. A novel approximation of an efficient adsorbent for As removal in contaminated water. J. Porous Mater..

[42] Chandrapati S., Williams M.G., Batt C.A., Tortorello M.L. (2014). Total viable counts|Most Probable Number (MPN). Encyclopedia of Food Microbiology.

[43] Grönemeyer J.L., Burbano C.S., Hurek T., Reinhold-Hurek B. (2012). Isolation and characterization of root-associated bacteria from agricultural crops in the Kavango region of Namibia. Plant Soil.

[44] Behera B.C., Yadav H., Singh S.K., Mishra R.R., Sethi B.K., Dutta S.K., Thaoi H.N. (2017). Phosphate solubilization and acidphosphatase activity of Serratia sp. isolated from mangrove soil of Mahanadi river delta, Odisha, India. J. Genet. Eng. Biotechnol..

[45] Bradford M.M. (1976). A rapid and sensitive method for the quantitation of microgram quantities of protein utilizing the principle of protein-dye binding. Anal. Biochem..

[46] R Core Team (2024). R: A Language and Environment for Statistical Computing.

[47] Chang C.H., Yang S.S. (2009). Thermo-tolerant phosphate-solubilizing microbes for multi-functional biofertilizer preparation. Bioresour. Technol..

[48] Zhu H.-J., Sun L.-F., Zhang Y.-F., Zhang X.-L., Qiao J.-J. (2012). Conversion of spent mushroom substrate to biofertilizer using a stress-tolerant phosphate-solubilizing Pichia farinose FL7. Bioresour. Technol..

[49] Kour D., Kour H., Khan S.S., Khan R.T., Bhardwaj M., Kailoo S., Kumari C., Rasool S., Yadav A.N., Sharma Y.P. (2023). Biodiversity and functional attributes of rhizospheric microbiomes: Potential tools for sustainable agriculture. Curr. Microbiol..

[50] Swain M.R., Laxminarayana K., Ray R.C. (2012). Phosphorus Solubilization by Thermotolerant *Bacillus subtilis* Isolated from Cow Dung Microflora. Agric. Res..

[51] Vaz-Moreira I., Silva M.E., Manaia C.M., Nunes O.C. (2008). Diversity of bacterial isolates from commercial and homemade composts. Microb. Ecol..

[52] Tortosa G., Fernández-González A.J., Lasa A.V., Aranda E., Torralbo F., González-Murua C., Fernández-López M., Benítez E., Bedmar E.J. (2021). Involvement of the metabolically active bacteria in the organic matter degradation during olive mill waste composting. Sci. Total. Environ..

[53] Li X., Li K., Wang Y., Huang Y., Yang H., Zhu P., Li Q. (2023). Diversity of lignocellulolytic functional genes and heterogeneity of thermophilic microbes during different wastes composting. Bioresour. Technol..

[54] Gajdács M., Urbán E., Stájer A., Baráth Z. (2021). Antimicrobial resistance in the context of the sustainable development goals: A brief review. Eur. J. Investig. Health Psychol. Educ..

[55] Bertelli C., Gray K.L., Woods N., Lim A.C., Tilley K.E., Winsor G.L., Brinkman F.S. (2022). Enabling genomic island prediction and comparison in multiple genomes to investigate bacterial evolution and outbreaks. Microb. Genom..

[56] Cavicchioli R., Ripple W.J., Timmis K.N., Azam F., Bakken L.R., Baylis M., Webster N.S. (2019). Scientists’ warning to humanity: Microorganisms and climate change. Nat. Rev. Microbiol..

[57] He Y., Xie K., Xu P., Huang X., Gu W., Zhang F., Tang S. (2013). Evolution of microbial community diversity and enzymatic activity during composting. Res. Microbiol..

[58] Liu J., Cade-Menun B.J., Yang J., Hu Y., Liu C.W., Tremblay J., LaForge K., Schellenberg M., Hamel C., Bainard L.D. (2018). Long-term land use affects phosphorus speciation and the composition of phosphorus cycling genes in agricultural soils. Front. Microbiol..

[59] Bhatia A., Rajpal A., Madan S., Kazmi A.A. (2015). Techniques to analyze microbial diversity during composting—A mini review. Indian J. Biotechnol..

[60] Nabika H., Unoura K. (2016). Interaction between nanoparticles and cell membrane. Surface Chemistry of Nanobiomaterials.

[61] Partanen P., Hultman J., Paulin L., Auvinen P., Romantschuk M. (2010). Bacterial diversity at different stages of the composting process. BMC Microbiol..

[62] Ma S., Fang C., Sun X., Han L., He X., Huang G. (2018). Bacterial community succession during pig manure and wheat straw aerobic composting covered with a semi-permeable membrane under slight positive pressure. Bioresour. Technol..

[63] Macaskie L.E., Bonthrone K.M., Rouch D.A. (1994). Phosphatase-mediated heavy metal accumulation by a *Citrobacter* sp. and related enterobacteria. FEMS Microbiol. Lett..

[64] Waithaisong K., Kliangpradith K., Punnasiri P., Thaingthaum P., Thongaram T., Romruen U., Sridang P. (2022). Insoluble phosphate solubilisation and acid phosphatase activity of bacteria isolated from organic paddy soils. Health Sci. Sci. Technol. Rev..

